# Integration of Glutamate Dehydrogenase and Nanoporous Gold for Electrochemical Detection of Glutamate

**DOI:** 10.3390/bios13121023

**Published:** 2023-12-10

**Authors:** Ting Cai, Keshuai Shang, Xiaolei Wang, Xiaoyan Qi, Ruijun Liu, Xia Wang

**Affiliations:** State Key Laboratory of Microbial Technology, Shandong University, Qingdao 266237, China; cait2023@163.com (T.C.); asakas13468242423@163.com (K.S.); wxl18354673483@163.com (X.W.); xyqi5028@163.com (X.Q.); 202332593@mail.sdu.edu.cn (R.L.)

**Keywords:** electrochemical detection, biosensor, glutamate dehydrogenase, nanostructured porous gold, glutamate

## Abstract

Glutamate, a non-essential amino acid produced by fermentation, plays a significant role in disease diagnosis and food safety. It is important to enable the real-time monitoring of glutamate concentration for human health and nutrition. Due to the challenges in directly performing electrochemical oxidation–reduction reactions of glutamate, this study leverages the synergistic effect of glutamate dehydrogenase (GLDH) and nanoporous gold (NPG) to achieve the indirect and accurate detection of glutamate within the range of 50 to 700 μM by measuring the generated quantity of NADH during the enzymatic reaction. The proposed biosensor demonstrates remarkable performance characteristics, including a detection sensitivity of 1.95 μA mM^−1^ and a limit of detection (LOD) of 6.82 μM. The anti-interference tests indicate an average recognition error ranging from −3.85% to +2.60%, spiked sample recovery rates between 95% and 105%, and a relative standard deviation (RSD) of less than 4.97% for three replicate experiments. Therefore, the GLDH-NPG/GCE biosensor presented in this work exhibits excellent accuracy and repeatability, providing a novel alternative for rapid glutamate detection. This research contributes significantly to enhancing the precise monitoring of glutamate concentration, thereby offering more effective guidance and control for human health and nutrition.

## 1. Introduction

As a fundamental amino acid involved in nitrogen metabolism and a crucial excitatory neurotransmitter in the central nervous system, the clinical detection of glutamate has great significance for hepatic diseases, pancreatic neoplasms, glutamate poisoning, and various neurological disorders [1,2,3,4]. Furthermore, glutamic acid has been widely employed in the production of condiments like monosodium glutamate, making it one of the most prolific amino acids [5,6]. It also plays a pivotal role in the food, cosmetics, and pharmaceutical industries [7,8]. Consequently, there is a compelling need to develop a simple, rapid, and highly sensitive method for detecting glutamate, which will be of great significance for the diagnosis of certain diseases and the composition monitoring of daily products.

The current technologies being used to measure glutamate include magnetic resonance spectroscopy (MRS) [9], capillary electrophoresis (CE) [10], high-performance liquid chromatography (HPLC) [11,12], and gas chromatography–mass spectrometry (GC-MS) [13,14]. Although these methods measure glutamate with high accuracy, they require expensive equipment, trained professionals, and complex operating procedures. The aforementioned intrinsic deficiencies render the approaches unsuitable for practical implementation in routine diagnostic settings. Electrochemical detection methods have garnered considerable attention due to their inherent advantages such as cost-effectiveness, exceptional sensitivity, and user-friendly operation [15]. However, unlike cysteine [16], tyrosine [17], and tryptophan [18], which are electroactive amino acids that readily undergo direct electrocatalytic redox reactions, glutamate poses challenges due to its resistance to such reactions [19]. Thus, most electrochemical sensing strategies necessitate additional reactions to convert glutamic acid into electroactive substances, thereby enabling indirect electrochemical detection through synergistic effects [12,20]. In addition, the concentration fluctuation range of glutamate in some actual production processes is relatively large. Therefore, the effective detection range of the constructed biosensor needs to be as wide as possible. For instance, during the fermentation process, the concentration of glutamate can be gradually increased from zero to a high value (such as 140 mM) with the extension of the fermentation time [21], which is a relatively wide concentration range.

The addition of enzymes as biorecognition components to electrochemical transducers has been shown to provide additional selectivity, visibly enhancing the sensor’s response signal [20,22]. Glutamate dehydrogenase (GLDH) is an enzyme that utilizes NADP^+^ or NAD^+^ as a coenzyme and exhibits specific catalytic activity in the reversible conversion between glutamate and α-ketoglutaric acid, as shown in Equation (1) [23]. By increasing the reactant concentration and adjusting the reaction temperature, pH, and other conditions, the equilibrium of the reaction can be favorably shifted towards glutamate oxidation, resulting in the generation of an electroactive substance, NADH [24,25]. This well-defined biochemical pathway represents a fundamental mechanism in the design and fabrication of sensors specifically tailored for glutamate detection.

L-Glutamate + H_2_O + NAD^+^ = α-Ketoglutaric acid + NH_4_^+^ + NADH (1)

**Figure 1:**
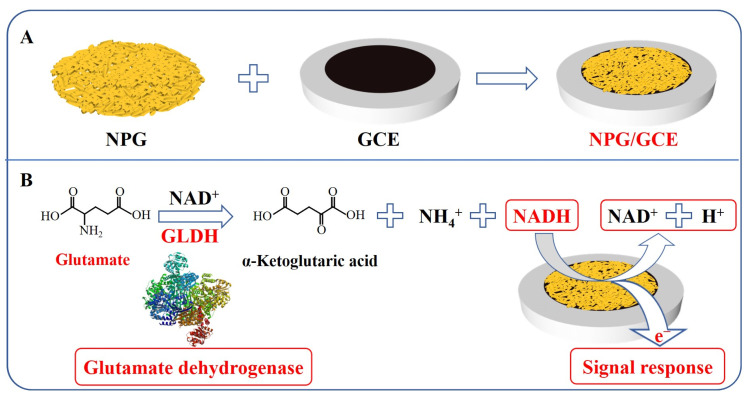
(**A**) The construction of NPG/GCE electrode. (**B**) The principle of indirect electrochemical detection of glutamate.

With the emergence of various novel materials, the performance of electrochemical sensors has been continuously improved [26,27]. Nanomaterials represented by NPG have been widely used as recognition elements of electrochemical sensors [28,29]. NPG possesses unique material properties that offer potential benefits for numerous applications, attributed to its high specific surface area, well-characterized gold mercaptan surface chemistry, high electrical conductivity, and reduced stiffness [30,31], which can significantly improve the sensitivity and signal-to-noise ratio of electrochemical sensors [32]. Owing to the exceptional biocompatibility and remarkable electrocatalytic properties exhibited by NPG for numerous compounds [33], this study meticulously opted for NPG as the modified material for the glass carbon electrode (GCE) to fabricate an NPG/GCE electrode (Figure 1A). The indirect detection of glutamic acid was accomplished through the precise response of the modified electrode to NADH, a byproduct of the enzymatic reaction (Figure 1B).

## 2. Materials and Methods

### 2.1. Materials and Equipment

Analytical-grade glutamic acid was purchased from Solarbio Technology Co., Ltd. (Beijing, China). Glutamate dehydrogenase with a specific activity of 268 U mg^−1^ was obtained from Yuan Ye Biotechnology Co., Ltd. (Shanghai, China). Nicotinamide adenine dinucleotide (NAD) with a purity higher than 98% was purchased from Aladdin Biochemical Technology Co., Ltd. (Shanghai, China). All other chemicals were of analytically pure grade. Deionized water was prepared using Millipore’s Direct-Q3 UV (Darmstadt, Germany) and used for all subsequent experiments. GCE, saturated calomel electrode (SCE), and α-Al_2_O_3_ powder were purchased from Aida Hengsheng Technology Development Co., Ltd. (Shanghai, China). The whole GCE was cylindrical, its inner core column was made of glass carbon, only the end was exposed (the radius is 2 mm), and the rest were wrapped with the insulating material, polytetrafluoroethylene. The CHI760E electrochemical workstation (Shanghai Chenhua Instrument Co., Ltd., Shanghai, China) was used for electrochemical measurements, as reported in previous studies [34].

### 2.2. Preparation of Electrodes

First, the GCE was sequentially polished with α-Al_2_O_3_ slurry of different particle sizes (1.0 µm, 0.3 µm, and 0.05 µm), followed by immersion in ultrapure water and anhydrous ethanol for ultrasonic cleaning. NPG films were prepared by dealloying Au/Ag sheet (Au50Ag50, wt%) in concentrated nitric acid at 40 °C for 1 h [35]. After covering the acid-etched NPG film onto the surface of the GCE (Figure 1A), an appropriate amount (3 μL) of Nafion solution (0.5 wt%) was introduced to enhance the stability of the NPG/GCE electrode assembly. Then, the NPG/GCE electrode was activated in H_2_SO_4_ (0.5 mol L^−1^) solution by cyclic voltammetry (CV). CV was repeatedly performed with a scanning rate of 50 mV s^−1^ within the voltage range of 0.35–1.55 V until a stable curve was obtained. All tests were conducted in a three-electrode system, utilizing the NPG/GCE electrode as the working electrode, the platinum as the counter electrode, and the SCE electrode as the reference electrode.

### 2.3. Feasibility Analysis of Indirect Electrochemical Detection of Glutamic Acid

To validate the viability of the proposed indirect electrochemical detection method for glutamic acid, the initial step involved investigating the electrochemical detection of NADH (Figure 1B). The CV technique was employed to examine the potential occurrence of an electrochemical catalytic reaction of NADH and assess its reaction characteristics. The NPG/GCE electrode was compared with an unmodified bare GCE electrode to ascertain the alterations in NADH sensing performance facilitated by NPG modification. Subsequently, CV detection was conducted on the enzymatic reaction solution containing various concentrations of glutamate to corroborate the accomplishment of electrochemical detection via the synergistic interaction between GLDH and NPG. By analyzing the CV curves derived from these experiments, it would be feasible to assess the viability and efficacy of the proposed indirect electrochemical detection scheme for glutamic acid utilizing NPG and NADH. The entire system was located in a phosphate-buffered solution (PBS, pH 7.0, 100 mM).

### 2.4. Optimization of Enzymatic Reactions

Single-factor experiments were employed to individually optimize the pH, temperature, and reaction time parameters of the enzymatic reaction system. The peak current signal obtained through differential pulse voltammetry (DPV) was utilized to ascertain the optimal conditions for NADH generation in the reversible reaction. Prior to accessing the DPV program, the final pH of the reaction system was uniformly adjusted to 7.0.

### 2.5. The Detection Performance and Anti-Interference Ability of Biosensor

Glutamate was incorporated into PBS (pH 7.0, 100 mM) at a final concentration range of 50 to 700 μM, along with 4.0 mM coenzyme NAD^+^ and 20 μL of GLDH (10 mg mL^−1^). The reaction was conducted under the pre-established optimal conditions. Upon completion, the DPV technique was employed for analysis. A linear regression analysis of the glutamate concentration against the corresponding peak current values was performed to construct a standard curve for the electrochemical detection of glutamate.

In order to assess the anti-interference ability of the proposed electrochemical sensing strategy for glutamate and explore its applicability in real scenarios, this study investigates the impact of common compounds and ions that could potentially act as interferents during the detection process of glutamate in actual samples, such as urine, serum, and fermentation broth. Under optimized conditions, DPV was employed to detect glutamate in a buffered solution with a final concentration of 500 μM. The effects of interferents, including potassium ions (K^+^), magnesium ions (Mg^2+^), hydrogen phosphate ions (HPO_4_^2−^), sulfate ions (SO_4_^2−^), glucose, urea, and uric acid, were systematically examined by analyzing the variations in the peak current resulted from NADH oxidation. This comprehensive investigation aimed to evaluate the robustness of the developed sensor against potential interferences.

## 3. Results and Discussion

### 3.1. Feasibility Verification of Glutamate Indirect Detection Sensor

**Figure 2:**
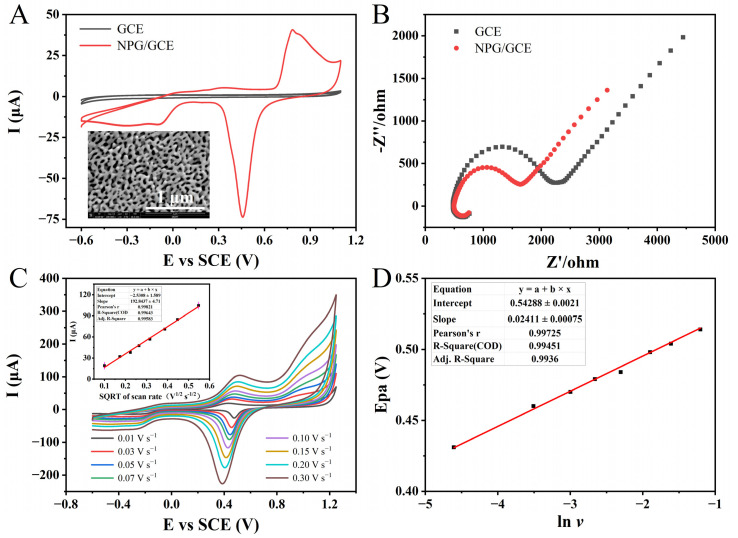
(**A**) The CVs of GCE and NPG/GCE in PBS (pH 7.0, 100 mM). Inset: SEM image of NPG at a magnification of 100,000×. (**B**) The EIS profiles of GCE and NPG/GCE in 5.0 mM [Fe(CN)_6_]^4−/3−^ solution. (**C**) The CVs of the NPG/GCE in PBS (pH 7.0, 100 mM) containing 3.0 mM NADH at different scan rates (0.01–0.30 V s^−1^). Inset: the fitting curve of the square root of oxidation peak current and scanning rate. (**D**) The fitting curve of peak oxidation potential and the natural logarithm of scanning rate.

Firstly, scanning electron microscopy (SEM) was employed for the morphological analysis of NPG films. The SEM imaging of the NPG film revealed that the NPG film, prepared through an advanced dealloying technique followed by a one-hour hot nitric acid treatment, exhibited a well-defined nanostructure consisting of nanoscale pores and interconnected metallic ribbons (Figure 2A). The average width of the metallic ribbons was approximately 30 nm, while the pore diameters varied within the range of a few nanometers to several tens of nanometers. In the investigation of the CO oxidation mechanism on NPG through the density functional theory (DFT), it was observed that the surface kinks joining the (111) and (100) planes of gold serve as the major active sites for catalytic activity [36]. This distinctive microstructural configuration endowed the NPG film with a significantly enhanced specific surface area, excellent structural stability, as well as abundant anchoring sites and active catalytic sites [37,38]. These features are crucial factors that contribute to the superior catalytic performance exhibited by NPG-modified electrodes compared to bare electrodes. The pretreated GCE electrode displayed a pristine, lustrous, and obsidian appearance, whereas the successfully assembled NPG/GCE electrode exhibited a uniformly deposited NPG film with a resplendent auric hue.

To verify the successful modification of NPG on the surface of GCE, CV characterization was performed on both activated GCE and NPG/GCE electrodes in PBS (pH 7.0, 100 mM). The results demonstrate that NPG/GCE exhibited well-defined redox peaks at an oxidation peak potential (*E_pa_*) of around +0.80 V and a reduction peak potential (*E_pc_*) of approximately +0.45 V in comparison to the unmodified bare GCE (Figure 2A). The above evidence confirmed the successful modification of NPG on the GCE electrode surface, and the reversible redox reaction of the “active” gold atoms in NPG under electrochemical catalysis. NPG demonstrated excellent electrochemical activity and conductivity, providing a solid foundation for subsequent experiments. Subsequently, electrochemical impedance spectroscopy (EIS) was employed to further characterize the GCE and NPG/GCE electrode in a 5.0 mM [Fe(CN)_6_]^4−/3−^ solution. The Nyquist plots depict the impedance spectra (Figure 2B). The large semicircle observed in the low-frequency region represents the mass transfer control, while the high-frequency region corresponds to the charge transfer control. The diameter of the semicircle in the high-frequency region is equivalent to the charge transfer resistance (Rct). This parameter reflects the Faradaic impedance characteristics of the sensing system, specifically indicating the efficiency of charge generation and transfer during the oxidation–reduction reactions of electroactive species at the electrode–electrolyte interface [39]. A larger semicircle diameter and charge transfer resistance indicate higher hindrances in the transport process, suggesting slower charge generation and lower transfer efficiency during the redox reactions of electroactive species [40]. The bare GCE electrode exhibited a larger semicircle, indicating a higher interfacial impedance. In contrast, the NPG/GCE electrode displayed a smaller semicircle, demonstrating enhanced electrical conductivity due to the successful modification with NPG. Electron transfer to the electrode surface was facilitated, and the rate of interface electron transfer was significantly increased.

In the presence of 3.0 mM of NADH in the PBS (pH 7.0, 100 mM), CV techniques were conducted using the NPG/GCE electrode at different scan rates (Figure 2C). Within the scanning potential range from −0.7 V to +1.2 V, a couple of asymmetrical redox peaks were obtained from the cyclic voltammetry. Moreover, with the escalation in the scanning rate, both the redox peak currents and redox potentials manifested noticeable alterations. The *E_pa_* exhibited a positive shift from +0.43 V at +0.01 V s^−1^ to +0.52 V at 0.3V s^−1^, indicating an increasing irreversibility of the electrode reaction. Simultaneously, the oxidation peak current increased from 19.00 μA to 104.50 μA. A fitting analysis revealed a positive correlation between the oxidation peak current and the square root of the scanning rate, described by the following linear regression equation: I (μA) = 192.8437 × *v* (V^1/2^ s^−1/2^) − 2.5308, (R^2^ = 0.9958). Consistent with previous research [41], the electrocatalytic oxidation of NADH on the surface of the NPG/GCE electrode exhibited a diffusion-controlled behavior. The fitting curve can prove that there is a linear relationship between the *E_pa_*(NADH) and the natural logarithm of the sweep rate, whose linear equation is *E_pa_*(NADH) = 0.02411 × ln*v* + 0.54288 (R^2^ = 0.9936) (Figure 2D). Based on the Laviron equation, the electron transfer number (n) during the process of NADH oxidation can be determined as 2 [42]. This result is consistent with the electron transfer stoichiometry observed in previous investigations of NADH oxidation [43].

The signal output of the envisioned glutamate sensor in this work is achieved through the indirect detection of NADH. The results mentioned above demonstrate that the superior electrochemical characteristics of the NPG/GCE electrode and the unique electrochemical behavior of NADH on the NPG/GCE electrode surface are sufficient to support the feasibility of the proposed concept.

### 3.2. Construction of Electrochemical Detection System for NADH

**Figure 3:**
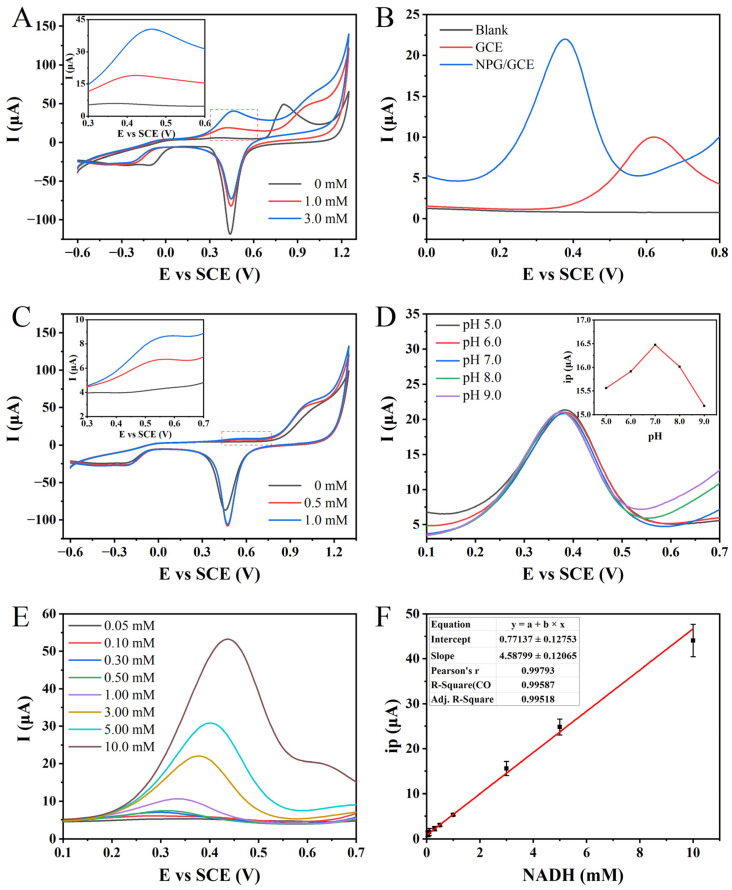
(**A**) The CVs of 0, 1.0, and 3.0 mM NADH detected by NPG/GCE in PBS (pH 7.0, 100 mM) at 50 mV s^−1^. (**B**) The DPVs of 0 mM and 3.0 mM NADH in PBS (pH 7.0, 100 mM) were detected by GCE and NPG/GCE electrodes. (**C**) The CVs of glutamate (0, 0.5, and 1.0 mM) were detected by NPG/GCE in PBS (pH 7.0, 100 mM) at 50 mV s^−1^. (**D**) The DPVs of NPG/GCE for 3.0 mM NADH at different pH values (5.0–9.0) in PBS (100 mM). Inset: the relationship between oxidation peak current (ip) and pH. (**E**) The DPVs of NPG/GCE electrode for 0.05–10.0 mM NADH in PBS (pH 7.0, 100 mM). (**F**) The fitting curve of ip and NADH concentration.

In order to establish the standard curve for the electrochemical detection of NADH, the electrochemical behavior of NADH and the relevant influencing factors in the detection process were further determined. A comparison between the CV curve obtained from the NPG/GCE electrode in blank PBS and the CVs measured at NADH concentrations of 1.0 mM and 3.0 mM revealed a distinct oxidation peak centered around +0.45 V (Figure 3A). Notably, the magnitude of the peak current associated with this oxidation peak increased proportionally with the NADH concentration. These findings provide evidence that NADH undergoes oxidation on the surface of the NPG/GCE electrode during CV analysis, leading to the generation of a prominent peak current signal. Importantly, no reduction peak corresponding to the reverse reaction of NADH is observed, indicating the irreversibility of the electrochemical process.

To validate the improvement in electrode sensitivity towards NADH due to NPG modification, the prepared NPG/GCE electrode was compared with the bare GCE electrode without modification (Figure 3B). Compared to the blank PBS, the bare GCE electrode exhibited a distinct oxidation peak at +0.62 V in the DPV curve in the presence of 3.0 mM NADH, with a peak current value of 8.85 μA. However, in the PBS containing the same concentration of NADH, the DPV curve of the NPG/GCE electrode showed a significant decrease in the oxidation peak potential of NADH to +0.38 V, accompanied by a remarkable increase in the peak current to 17.28 μA. It is noteworthy that the oxidation potential decreased by 39.35%, while the peak current increased by 95.20% after the NPG modification. These findings conclusively demonstrate the effective enhancement of electrocatalytic activity and electron transfer efficiency towards NADH upon NPG modification.

Based on the confirmation that the NPG/GCE electrode enables the electrochemical-sensitive detection of NADH, the CV technique was employed to investigate the feasibility of the electrochemical detection of glutamate through the synergistic action of GLDH and NPG. Compared to the CV curve in blank PBS, distinct oxidation peaks were observed around +0.46 V when glutamate concentrations of 0.5 mM and 1.0 mM were tested (Figure 3C). These oxidation peaks coincide with the peaks obtained from the direct electrochemical detection of NADH (Figure 3A). Moreover, the peak currents also increase with the increasing glutamate concentration, indicating the viability of the indirect electrochemical detection approach for glutamate.

The pH of the electrolyte solution has a significant impact on the detection of electrochemically active substrates [44]. To improve the sensing performance and achieve the sensitive detection of glutamate, a detailed study was conducted using the DPV technique to investigate the peak current response of 3.0 mM of NADH in PBS with varying pH values. An intriguing trend was observed whereby the oxidation peak current of NADH exhibited a gradual increase with an elevation in the pH (Figure 3D). The highest peak current was attained at a pH value of 7.0, serving as the optimal point. However, as the pH value continued to rise beyond this point, a gradual decline in the peak current was witnessed. Consequently, it was concluded that adopting a PBS solution with a precisely regulated pH of 7.0 (100 mM) would serve as the optimal electrolyte medium for the efficient electrochemical detection of NADH, as well as for subsequent enzymatic reactions in the context of our experimental endeavors.

Based on the optimization results and electrochemical behavior analysis, a standard curve for NADH electrochemical detection was established using the NPG/GCE electrode utilizing the DPV technique. The peak current signal of NADH oxidation increased gradually with an increase in its concentration, while the peak potential exhibited a slight positive shift with an increasing concentration (Figure 3E). Subsequently, a linear fitting was conducted to establish the relationship between the oxidation peak current (ip) of NADH and its concentration. An excellent linear relationship was observed between the NADH concentration in the range of 0.05 to 10.0 mM, with a linear equation of ip (μA) = 4.58799 × *C_NADH_* (mM) + 0.77137 (R^2^ = 0.9952) (Figure 3F). The sensitivity was determined to be 4.50 μA mM^−1^, and the LOD was 2.92 μM (S/N = 3). These results indicate that the NPG/GCE electrode exhibits excellent NADH detection performance.

### 3.3. Optimization of Glutamate Dehydrogenase Reaction

**Figure 4:**
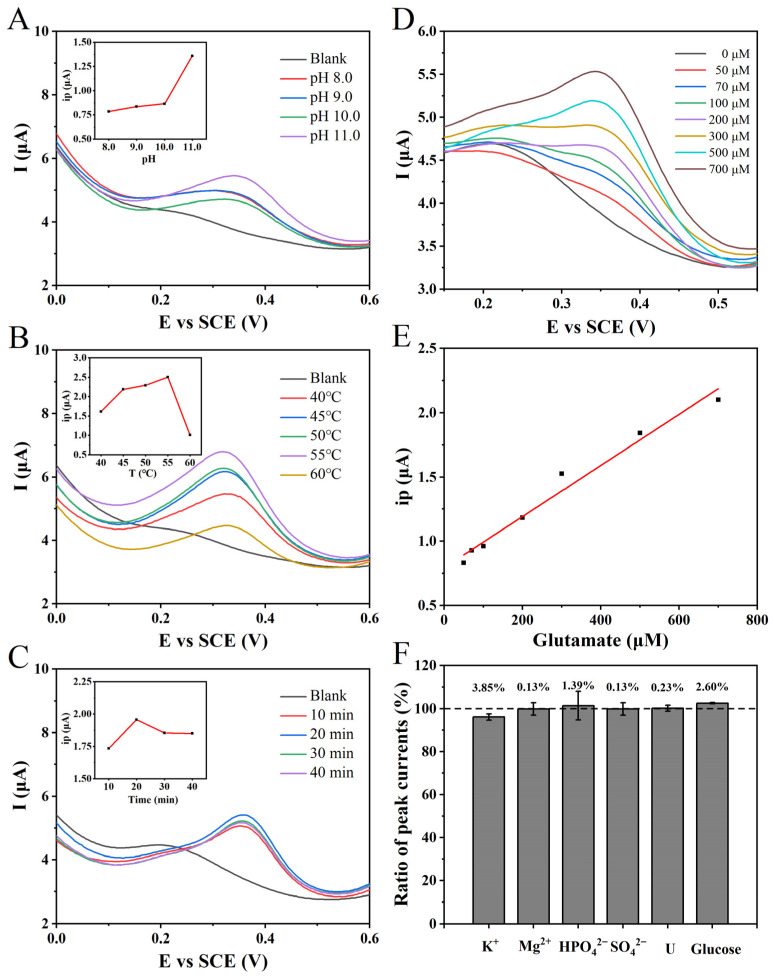
(**A**) The DPVs of the NPG/GCE for the reaction of 1.0 mM glutamate in PBS (100 mM) with different pH values (8.0–11.0) for 20 min. Inset: the relationship between the oxidation peak current and the pH value of the enzyme reaction. (**B**) The DPVs of the NPG/GCE electrode reacted with 1.0 mM glutamate in PBS (pH11.0, 100 mM) at different temperatures (40–60 °C) for 20 min. Inset: the relationship between the measured oxidation peak current and the enzyme reaction temperature. (**C**) The DPVs of 2.0 mM glutamate with NPG/GCE electrode after reaction in PBS (pH 11.0, 100 mM) and 55 °C for 10–40 min. Inset: the relationship between the measured oxidation peak current and the enzyme reaction temperature. (**D**) The DPVs of 0–700 μM glutamate by NPG/GCE electrode after reaction in PBS (pH 11.0, 100 mM) and 55 °C for 20 min. (**E**) The fitting relationship between the measured oxidation peak current value (ip) and glutamate concentration. (**F**) The effect of common substances (5.0 mM) in clinical samples and fermentation broth samples on glutamate (500 μM) detection.

To optimize the enzymatic activity of GLDH towards oxidation and to enhance the production of NADH at equilibrium, thereby improving the electrochemical sensing performance for glutamate, a systematic investigation was conducted on the influence of the reaction pH. While maintaining a consistent reaction temperature and duration, a 1.0 mM glutamate solution was subjected to reactions in PBS at different pH values ranging from 8.0 to 11.0. The DPV results demonstrated a progressive increase in the measured oxidation peak current with rising pH values (Figure 4A). This observation unequivocally establishes the optimal pH value for the maximum enzymatic activity of GLDH in the oxidation direction to be 11.0. Consequently, all subsequent experimental reactions were performed at pH 11.0 to ensure consistency and comparability.

Upon achieving pH optimization, a subsequent adjustment of the enzymatic reaction temperature was undertaken. With the pH and reaction time maintained at consistent levels, a 1.0 mM glutamate solution was subjected to a range of temperatures. The resulting DPV revealed that the highest oxidation peak current was recorded at a reaction temperature of 55 °C (Figure 4B). This observation signifies the maximal enzymatic activity of GLDH in the oxidation direction under these conditions. Therefore, all subsequent experimental enzymatic reactions were meticulously conducted at a precisely controlled temperature of 55 °C to ensure methodological rigor and enable accurate comparisons.

Under precisely optimized conditions of a reaction pH and temperature, a 2.0 mM glutamate solution in PBS (pH 11.0, 100 mM) was subjected to varying reaction durations, and the resulting DPVs are presented in Figure 4C. It was observed that as the reaction time increased, the measured oxidation peak current of NADH exhibited a gradual increment. The maximum value was achieved at a reaction time of 20 min. Subsequently, a slight decrease in the NADH peak current was observed with the further elongation of the reaction time, possibly due to the minor decomposition of NADH. Therefore, based on these findings, an optimal duration of 20 min was ascertained.

### 3.4. Glutamate Indirect Detection by GLDH-NPG/GCE Bioelectrode

Based on the optimization of NADH electrochemical detection and GLDH reaction conditions, a standard curve for the indirect electrochemical detection of glutamate was established using the constructed biosensor. Different concentrations of glutamate, 4.0 mM NAD^+^, and 20 μL of GLDH were added to PBS (pH 11.0, 100 mM), followed by an incubation at 55 °C for 20 min. The pH was subsequently adjusted to 7.0, and electrochemical detection was performed using the NPG/GCE electrode with the application of DPV. The experimental results exhibited an increasing trend in the oxidation peak current signal at approximately +0.40 V with escalating glutamate concentrations (Figure 4D). A linear regression analysis was then conducted to determine the relationship between the oxidation peak current signal and the glutamate concentration. According to the analysis findings, within the concentration range of 50 to 700 μM, a strong linear correlation was observed, characterized by the following linear equation: ip (μA) = 0.00195 × *C_Glu_* (μM) + 0.73386 (R^2^ = 0.9976) (Figure 4E). The proposed glutamate electrochemical sensing strategy demonstrated a detection sensitivity of 1.95 μA mM^−1^ and an LOD of 6.82 μM (S/N = 3), indicating its capacity for highly sensitive detection.

A comparative analysis was conducted between the glutamate electrochemical detection sensor developed in this study and previously reported sensors, as depicted in Table 1. The distinctive nanoporous structure of the NPG endowed it with a large surface area and abundant active sites, resulting in remarkable NADH electrocatalytic activity for the NPG/GCE electrode. The direct electrochemical detection of NADH exhibited a broad linear range of 0.05 to 10 mM concentration, displaying excellent linearity and a relatively low detection limit. The proposed method for glutamate electrochemical detection demonstrated a wide linear range (50–700 μM), meeting the practical detection requirements. Moreover, the biosensor offers notable advantages such as stability, cost-effectiveness, and straightforward fabrication processes, indicating substantial potential for practical applications.

### 3.5. Selectivity and Anti-Interference of GLDH-NPG/GCE Biosensor

The performance evaluation of biosensors, designed for the specific identification of target analytes in practical applications, primarily depends on two crucial factors: selectivity and anti-interference. To assess the anti-interference capability of the proposed glutamate electrochemical sensing strategy, various common nitrogen sources, carbon source compounds, and ions were individually added to a PBS solution (pH 11.0, 100 mM) containing 500 μM glutamate. The concentration of interfering substances was standardized to 5.0 mM. After the enzymatic reaction, the relative peak current signal ratios were measured using the DPV method compared to the control group without interference substances. The results indicate that the addition of interferents had a minimal impact on the indirect electrochemical detection of glutamate, with relative peak current variations ranging from −3.85% to +2.60% (Figure 4F). These findings demonstrate that the presence of the added interferents does not affect the detection of glutamate, highlighting the excellent anti-interference and selectivity of the glutamate detection biosensor. This further suggests the potential for glutamate detection in real samples such as fermentation broth and its promising application prospects.

### 3.6. Actual Sample Testing of GLDH-NPG/GCE Biosensor

Building upon the investigation of anti-interference capability, the performance and practical application potential of the constructed GLDH-NPG/GCE biosensor was explored through spike-in recovery experiments using fermentation broth. The results, as presented in Table 2, demonstrate the recoveries of the spiked glutamate rates ranging from 95% to 105%, with a relative standard deviation (RSD) below 4.97% for three repeated experiments. Furthermore, it was observed that the GLDH-NPG/GCE biosensor exhibits enhanced detection accuracy under lower glutamate concentration conditions compared to higher concentrations. Specifically, when spiked with 100 μM of glutamate, the RSD is as low as 0.38%. However, as the spiked concentration increases to 700 μM, the RSD also increases to 4.97%. Consequently, it can be inferred that the sensor yields more dependable detection outcomes for samples characterized by relatively lower glutamate concentrations (100 μM). In conclusion, the constructed GLDH-NPG/GCE electrochemical sensor exhibits exceptional accuracy and repeatability for glutamate detection, endorsing the proposed glutamate electrochemical sensing strategy as being highly promising for a practical sample analysis.

## 4. Conclusions

Based on the excellent electrocatalytic activity exhibited by NPG towards various compounds, a novel electrochemical sensing strategy for the indirect detection of glutamate was developed through the synergistic action of glutamate dehydrogenase (GLDH) and an NPG-modified glassy carbon electrode (NPG/GCE). The constructed GLDH-NPG/GCE biosensor demonstrates exceptional accuracy and repeatability in detecting glutamate through selective NADH recognition. Within the detection range of 50–700 μM, a good linear correlation exists between the oxidation peak current and glutamate concentration, with a high sensitivity of 1.95 μA mM^−1^ and a low detection limit of 6.82 μM. In addition, the sensor also has strong anti-interference performance and practical application potential. The observations highlight the commendable accuracy and repeatability exhibited by the GLDH-NPG/GCE biosensor. In conclusion, the GLDH-NPG/GCE synergistic electrochemical sensor developed in this study enables the rapid and sensitive detection of glutamate, showing promising prospects for practical applications.

## Figures and Tables

**Table 1:** Performance comparison between glutamate detection sensors.

Electrode Modification	Method	Detection Range (μM)	LOD (μM)	Reference
GluO_x_/[C_3_(OH)_2_mim] [BF_4_]/Au-Pt NPs/Nafion	IT	0.5–20	0.17	[45]
Pt-disc/PEI/GlutOx/PPD-BSA	IT	5–50	2.5	[46]
MWCNT-MB/GLDH-NAD/MWCNT-CHIT	IT	7.5–105	3	[47]
GluOx/Co_3_O_4_/CS/GR/GCE	IT	4–600	2	[48]
GLDH-NPG/GCE	DPV	50–700	6.82	This work

**Table 2:** The spike recovery of glutamate detection in actual samples.

Samples	Spiked Glutamate (μΜ)	Detected by NPG/GCE (μΜ)	Recovery Rate (%)	RSD (%)
1	100.00	104.06 ± 0.39	104.06	0.38
2	400.00	419.89 ± 5.50	104.97	1.31
3	700.00	719.06 ± 35.75	102.72	4.97

## Data Availability

The data are contained within the article.
